# Green tea consumption and lung cancer risk: the Ohsaki study

**DOI:** 10.1038/sj.bjc.6604645

**Published:** 2008-09-02

**Authors:** Q Li, M Kakizaki, S Kuriyama, T Sone, H Yan, N Nakaya, K Mastuda-Ohmori, I Tsuji

**Affiliations:** 1 Division of Epidemiology, Department of Public Health and Forensic Medicine, Tohoku University Graduate School of Medicine, Sendai 980-8575, Japan; 2 Division of Epidemiology and Biostatistics, Department of Public Health, Xi’an Jiaotong University School of Medicine, Xi’an 710061, China

**Keywords:** green tea, lung cancer, prospective cohort studies, Japan

## Abstract

We examined the risk of lung cancer in relation to green tea consumption in a population-based cohort study in Japan among 41 440 men and women, aged 40–79 years, who completed a questionnaire in 1994 regarding green tea consumption and other health-related lifestyle factors. During the follow-up period of 7 years (from 1995 to 2001), 302 cases of lung cancer were identified, and the Cox proportional hazards regression model was used to estimate the hazard ratios (HRs) and 95% confidence intervals (CIs). The multivariable-adjusted HRs of lung cancer incidence for green tea consumption of 1 or 2, 3 or 4, and 5 or more cups/day as compared to less than 1 cup/day were 1.14 (95% CI: 0.80–1.62), 1.18 (95% CI: 0.83–1.66), and 1.17 (95% CI: 0.85–1.61), respectively (*P* for trend=0.48). This cohort study has found no evidence that green tea consumption is associated with lung cancer.

Lung cancer is the commonest cause of cancer death worldwide (Parkin, 2001; Parkin *et al*, 2005) and now ranks first among cancers in Japan. In 2005, lung cancer deaths accounted for approximately 19% of all cancer deaths in Japan (23% in men and 13% in women, The Editorial Board of The Cancer Statistics in Japan, 1952–2005).

Evidence from *in vitro* and animal experiments has suggested that green tea polyphenols protect against lung cancer through their antimutagenic and antioxidant properties (Valcic *et al*, 1996; Okabe *et al*, 1997; Yang *et al*, 1998, 2005; Fujimoto *et al*, 2002; Chen *et al*, 2004; Kuzuhara *et al*, 2007; Sadava *et al*, 2007). To the best of our knowledge, only two studies (of atomic bomb survivors and postmenopausal women) have investigated lung cancer risk in relation to green tea or total tea consumption (Zheng *et al*, 1996; Nagano *et al*, 2001), and both found no association.

In a population-based prospective study of men and women in Miyagi Prefecture in northeastern Japan, where about 75% of the population drinks green tea everyday (Suzuki *et al*, 2004), we investigated further whether an association exists between green tea consumption and lung cancer risk.

## Materials and methods

The details of this Ohsaki National Health Insurance (NHI) cohort study have been published elsewhere (Tsuji *et al*, 1998; Kikuchi *et al*, 2006; Kuriyama *et al*, 2006; Nakaya *et al*, 2007). In brief, a baseline self-administered questionnaire was distributed to all NHI beneficiaries aged 40–79 years living in the catchment area of the Ohsaki Public Health Center, Miyagi Prefecture, in northeastern Japan from October to December 1994. From 54 996 eligible individuals, 52 029 (94.6%) individuals responded to the survey. We considered the return of self-administered questionnaires signed by the participants to imply their consent to participate in the study. The study protocol was approved by the institutional review board of the Tohoku University Graduate School of Medicine.

We excluded 776 individuals who had withdrawn from the NHI before we started the prospective study, 3038 individuals with a prior history of cancer, 6355 individuals with missing data on green tea consumption, and 420 individuals who had reported extreme daily energy intake (outside of the sex-specific cutoff points of lower 0.5% and upper 0.5%). Finally, 41 440 individuals were involved in the final analysis.

The baseline survey questionnaire contained items on basic personal health information and a food frequency questionnaire (FFQ). Measurements of cigarette smoking included smoking status (never, current, or past), number of cigarettes smoked per day, and total years of cigarettes smoked. Five frequency categories were used for the food consumption as almost never, 1–2 times/month, 1–2 times/week, 3–4 times/week, and almost every day. For green tea and coffee, the five categories were almost never, occasionally, 1–2 cups/day, 3–4 cups/day, and more than 5 cups/day. We combined the first two categories of green tea consumption into a single category as less than 1 cup/day because very few individuals selected those options. The validity of the FFQ was assessed by calculating Spearman's correlation coefficients between four 3-day diet records collected during four seasons within the year and the reproducibility evaluated by two FFQ 1 year apart. The results showed a reasonably high level of validity and reproducibility. The corresponding Spearman's correlation coefficients between the diet records and FFQ on green tea for men and women are 0.71 and 0.53, between the two FFQ are 0.63 for men and 0.64 for women, respectively (Ogawa *et al*, 2003).

The end point in this study was lung cancer incidence defined as code C34.0–C34.9, according to the International Classification of Diseases for Oncology, second edition (ICD-O-2) (Percy *et al*, 1990). We ascertained the incidence of lung cancer through a computerised record linkage to the Miyagi Prefecture Cancer Registry, one of the oldest and most accurate population-based cancer registries in Japan (Takano and Okuno, 1997). We followed incidence of cancer, death, emigration, or loss of NHI qualification of each study individual by obtaining their NHI claim history files from the Miyagi NHI Association. The date and reasons of withdrawal were obtained from the records of the NHI withdrawal history files. Follow-up of the individuals who had withdrawn from the NHI was discontinued because of logistic limitations.

### Statistical analysis

We counted the person-years of follow-up for each individual from 1 January 1995 until the date of diagnosis of lung cancer, date of withdrawal from the NHI, date of death, or the end of the study period (31 December 2001), whichever occurred first. We used the Cox proportional hazards regression to estimate the hazard ratios (HRs) and 95% confidence intervals (CIs) of lung cancer incidence according to the categories of green tea consumption, using the SAS software, version 9.1 (SAS Institute Inc., Cary, NC, USA). The *P*-values for the test of linear trend were calculated by treating the green tea consumption category as a continuous variable. All reported *P*-values are two-side and the statistically significant cut point is less than 0.05.

We considered the following variables as potential confounders a priori: age at baseline (continuous variable), sex (men or women), education level (junior high school or lower, high school or higher), marital status (married, or widowed/divorced/single), body mass index (calculated as weight in kilograms divided by height in metres squared; <18.5, 18.5–24.9, or ⩾25.0), time spent walking (<1 or ⩾1 h day^−1^), family history of cancer (yes or no), smoking status (never, former, or current), number of cigarettes smoked per day (continuous variable), years of smoking (continuous variable), passive smoking (yes or no), alcohol drinking (never, former, current ethanol drinking of <45.6 g day^−1^, or current ethanol drinking of ⩾45.6 g day^−1^), daily total energy intake (continuous variable), daily consumption of soybean products, total meat, total fish, dairy products, total fruits, total vegetables (each food group was calculated by combining the individual food item and treat as a continuous variable), and consumption of coffee (never or occasionally, 1–2 cups/day, or ⩾3 cups/day). The age and sex adjusted model, age, sex, and smoking adjusted model were also computed and compared with the multivariable-adjusted model. The interactions between green tea consumption and confounders were tested through the addition of cross-product terms to the multivariable-adjusted model.

## Results

The baseline characteristics of the study individuals according to green tea consumption are shown in Table 1. Women tended to drink more green tea than men. The frequency of green tea consumption increased with mean age at baseline. Individuals who drank more green tea tended to consume more foods such as total fish, total fruits, total vegetables, dairy products, soybean products, as well as total energy. Green tea consumption was not related to total meat consumption. Fewer daily coffee drinkers (⩾3 cups/day) and alcohol drinkers were found in the high tea consumption group. Other potential factors were unrelated to green tea consumption, except for a moderate increase in the proportion of individuals with family history of cancer.

During the 7-year follow-up (until 31 December 2001), 302 cases of lung cancer were documented and 4928 individuals who withdrew from the NHI were loss to follow-up. Table 2 shows details of green tea consumption and lung cancer risk by the HRs and 95% CIs. We found no association between green tea consumption and lung cancer risk in women but a significant positive association in men in the univariate model with no adjustment of confounders, but this disappeared when age was taken into account. The multivariable-adjusted HRs as compared to none or less than 1 cup of green tea consumption per day were 1.14 (95% CI: 0.80–1.62) for 1–2 cups/day, 1.18 (95% CI: 0.83–1.66) for 3–4 cups/day, and 1.17 (95% CI: 0.85–1.61) for 5 or more cups/day (*P* for trend=0.48). The results were similar for men and women. The comparison between the age and sex adjusted model, the age, sex, and smoking adjusted model, and the different multivariable-adjusted model suggested that the estimates were stable. The findings did not change substantially after the lung cancer cases diagnosed in the first 3 years of the follow-up were excluded to avoid any bias of diet or lifestyle changes from undiagnosed lung cancer present at baseline (data not shown).

When the data were stratified according to age, smoking status, or other variables in the different categories, the results did not change. Data in the subgroup did not show any significant associations between lung cancer risk and green tea consumption (Table 3). The multivariable-adjusted HRs for 1–2 cups/day, 3–4 cups/day, and 5 or more cups/day of green tea consumption when compared with less than 1 cup/day were 1.07 (95% CI: 0.64–1.77), 1.41 (95% CI: 0.88–2.47), and 1.33 (95% CI: 0.85–2.07) respectively, for current smokers (*P* for trend=0.15). The corresponding data for never smokers were 1.59 (95% CI: 0.68–3.69), 1.41 (95% CI: 0.61–3.26), and 1.24 (95% CI: 0.54–2.84), respectively (*P* for trend=0.82). No significant interaction was observed in an additional analysis for interactive effects between tea intake and the corresponding confounders (data not shown).

## Discussion

In this large population-based prospective cohort study, we found no association between green tea consumption and the risk of lung cancer among this Japanese population, who consume green tea much more frequently than people in Western countries. There was no indication that green tea reduces lung cancer risk among past smokers, current smokers, or passive smokers.

It has been hypothesised that the consumption of green tea may decrease the risk of lung cancer risk, specifically in some case–control studies (Ohno *et al*, 1995; Mendilaharsu *et al*, 1998; Zhong *et al*, 2001; Hu *et al*, 2002). However, other case–control studies found either no association (Le Marchand *et al*, 2000; Bonner *et al*, 2005) or a positive association (Tewes *et al*, 1990). The wide range and crude categorisation of tea consumption, different study populations, choice of controls, inadequate control for confounding, and inevitable recall bias might have obscured possible relationships. Our findings are in general agreement with those of the prospective cohort study of atomic bomb survivors in Japan (Nagano *et al*, 2001) and postmenopausal women in America (Zheng *et al*, 1996), although tea consumption was differently categorised.

Our study had several strengths. Our individuals were recruited from the general population. The information on green tea consumption and other health-related lifestyle factors was obtained before the individuals developed lung cancer, thus avoiding recall bias. The questionnaire used had been tested and a reasonably high level of validity and reproducibility was found. Our sample size was appropriate given the high incidence in the study region, and the period of follow-up was reasonably long.

Our study also had some limitations. First, the information on green tea consumption and other lifestyle factors was collected only once. Some individuals might have changed their frequency of tea consumption during the follow-up so some misclassification was inevitable. Second, we excluded 6355 individuals who had not answered the question on green tea consumption and 420 individuals who had reported extreme daily energy intake, among whom 52 lung cancers were diagnosed. The characteristics of individuals without tea consumption details similar to those with mean age (61.8 *vs* 59.9), percentage of men (51.3 *vs* 47.3), and percentage of current smokers (29.4 *vs* 28.4). There was no difference in lung cancer incidence between the individuals included (*n*=41 440) and those excluded from the analysis (*n*=6775), and the HR was 1.03 (95% CI: 0.77–1.37; *P=*0.85). Thus, our results would not be substantially biased by exclusion of the individuals without tea data. The third concern is the 11.9% (4928 individuals) of participants lost to follow-up, but their characteristics were similar to those who were fully followed-up (*n*=36 512) with respect to: mean age (59.6 *vs* 58.0), percentage of men (47.3 *vs* 39.6), % of current smokers (30.6 *vs* 28.9), and percentage of individuals consuming more than 5 cups of green tea per day (30.8 *vs* 26.1). Thus, our results would not be remarkably biased by loss to follow-up.

This large population-based prospective cohort study in Japan finds no evidence that the consumption of green tea reduces lung cancer incidence.

## Figures and Tables

**Table 1:** Baseline characteristics of individuals according to green tea consumption, the Ohsaki study

	**Green tea consumption, cups/day**			
	**<1**	**1 or 2**	**3 or 4**	**⩾5**
**Characteristics**	**(*n*=10950)**	**(*n*=8983)**	**(*n*=9024)**	**(*n*=12 483)**
Age in years, mean (s.d.)	58.0 (10.8)	58.7 (10.8)	60.7 (10.0)	61.9 (9.5)
				
*Sex, no. (%)*				
Men	5959 (54.4)	4447 (49.5)	3998 (44.3)	5185 (41.5)
Women	4991 (45.6)	4536 (50.5)	5026 (55.7)	7298 (58.5)
				
*Smoking status, no. (%)*				
Never	4634 (49.2)	4085 (53.4)	4459 (57.8)	5965 (56.7)^a^
Former	1521 (16.1)	1116 (14.6)	1161 (15.0)	1551 (14.7)
Current, <20 cigarettes/day	1190 (12.6)	869 (11.4)	803 (10.4)	1226 (11.7)
Current, ⩾20 cigarettes/day	2080 (22.1)	1578 (20.6)	1296 (16.8)	1786 (17.0)
				
*Passive smoking, no. (%)*				
Yes	4190 (48.9)	3306 (49.0)	2942 (45.9)	4241 (49.5)
No	4378 (51.1)	3444 (51.0)	3467 (54.1)	4323 (50.5)
				
*Alcohol drinking, no. (%)*				
Never	3910 (40.2)	3371 (42.6)	3749 (47.1)	5297 (48.7)
Former	838 (8.6)	576 (7.3)	560 (7.0)	844 (7.8)
Current, <45.6 g day^−1^ ethanol	2996 (30.8)	2506 (31.6)	2355 (29.6)	3151 (29.0)
Current, ⩾45.6 g day^−1^ ethanol	1984 (20.4)	1470 (18.6)	1301 (16.3)	1592 (14.6)
				
*Education level, no. (%)*				
Junior high school or lower	6306 (61.0)	4783 (55.8)	4855 (56.1)	7145 (59.7)
High school	3298 (31.9)	3055 (35.6)	3027 (35.0)	3841 (32.1)
College/university or higher	739 (7.1)	733 (8.6)	768 (8.9)	980 (8.2)
				
*Walking duration, h day* ^*−1*^ *, no. (%)*				
<1	5277 (52.9)	4507 (54.3)	4761 (57.2)	6377 (55.7)
⩾1	4706 (47.1)	3794 (45.7)	3556 (42.8)	5076 (44.3)
				
*Body mass index, no. (%)* b				
<18.5	348 (3.4)	255 (3.0)	259 (3.0)	368 (3.1)
18.5–24.9	6914 (67.7)	5807 (68.4)	5884 (68.7)	7918 (66.9)
⩾25	2950 (28.9)	2430 (28.6)	2421 (28.3)	3558 (30.0)
				
*Marital status, no. (%)*				
Married	8027 (81.7)	6788 (82.8)	6831 (82.2)	9235 (80.6)
Widowed/divorced	1276 (13.0)	1063 (13.0)	1206 (14.5)	1899 (16.6)
Single	525 (5.3)	350 (4.3)	275 (3.3)	319 (2.8)
				
*Family history of cancer, no. (%)*				
Yes	3139 (28.7)	2667 (29.7)	2949 (32.7)	4230 (33.9)
No	7811 (71.3)	6316 (70.3)	6075 (67.3)	8253 (66.1)
				
Total energy intake, mean (s.d.), kcal day^−1^	1507 (594)	1516 (569)	1524 (544)	1554 (542)
				
*Daily dietary consumption*				
Soybean products, mean (s.d.), g	44.7 (26.8)	48.5 (25.9)	50.9 (24.7)	53.5 (24.1)
Total meat, mean (s.d.), g	17.8 (16.0)	18.1 (15.2)	17.8 (14.2)	17.6 (14.9)
Total fish, mean (s.d.), g	53.7 (34.6)	56.1 (33.9)	59.2 (32.6)	63.4 (33.2)
Dairy products, mean (s.d.), g	130.0 (101.1)	139.9 (100.7)	145.7 (100.3)	147.1 (101.3)
Total fruits, mean (s.d.), g	78.9 (61.2)	90.6 (62.8)	101.0 (63.1)	111.6 (63.9)
Total vegetables, mean (s.d.), g	84.6 (54.7)	94.9 (55.6)	102.1 (54.8)	108.6 (57.1)
Coffee, ⩾3 cups, no. (%)	1338 (13.5)	862 (11.3)	695 (9.2)	897 (8.8)

s.d.=standard deviation.

a The total percentage does not equal to 100 because of rounding, all the same.

b Body mass index was calculated as weight in kilograms divided by height in metres squared.

**Table 2:** Hazard ratios and 95% confidence intervals for lung cancer incidence according to green tea consumption category, the Ohsaki study

	**Green tea consumption, cups/day**								
	**<1**		**1 or 2**		**3 or 4**		**⩾5**		
**Characteristics**	**No.**	**HR**	**No.**	**HR (95% CI)**	**No.**	**HR (95% CI)**	**No.**	**HR (95% CI)**	***P* for trend^a^**
*All participants*									
Person-years of follow-up	66 894		55 390		56 365		78 627		
No. of lung cancer	66		61		67		108		
Crude		1.00		1.12 (0.79–1.58)		1.20 (0.86–1.69)		1.39 (1.02–1.89)	0.03
Age and sex-adjusted		1.00		1.14 (0.81–1.62)		1.16 (0.82–1.63)		1.28 (0.94–1.75)	0.12
Age, sex, smoke-adjusted		1.00		1.13 (0.80–1.60)		1.15 (0.82–1.62)		1.16 (0.85–1.59)	0.40
Multivariate adjusted^b^		1.00		1.14 (0.80–1.62)		1.18 (0.83–1.66)		1.17 (0.85–1.61)	0.48
									
*Men*									
Person-years of follow-up	36 798		27 516		24 880		32 653		
No. of lung cancer	54		43		51		79		
Crude		1.00		1.07 (0.71–1.59)		1.40 (0.95–2.05)		1.65 (1.17–2.33)	<0.01
Age adjusted		1.00		1.04 (0.70–1.55)		1.17 (0.80–1.72)		1.27 (0.90–1.80)	0.41
Age and smoke-adjusted		1.00		1.01 (0.68–1.50)		1.16 (0.79–1.70)		1.12 (0.79–1.59)	0.43
Multivariate adjusted^b^		1.00		1.05 (0.70–1.57)		1.21 (0.82–1.79)		1.17 (0.82–1.68)	0.32
									
*Women*									
Person-years of follow-up	30 096		27 873^c^		31 485		45 974		
No. of lung cancer	12		18		16		29		
Crude		1.00		1.62 (0.78–3.36)		1.27 (0.60–2.68)		1.57 (0.80–3.08)	0.31
Age adjusted		1.00		1.52 (0.73–3.15)		1.12 (0.53–2.37)		1.33 (0.68–2.61)	0.65
Age and smoke-adjusted		1.00		1.55 (0.75–3.22)		1.16 (0.55–2.45)		1.32 (0.67–2.58)	0.68
Multivariate adjusted^b^		1.00		1.48 (0.71–3.10)		1.11 (0.52–2.37)		1.30 (0.65–2.60)	0.71

HR=hazard ratio; CI=confidence interval.

a The *P*-values for the test of linear trend were calculated by treating the green tea consumption category as a continuous variable.

b The multivariate HR has been adjusted for age (continuous variable), sex (men or women, among ‘all participants’), education level (junior high school or lower, high school, or college/university or higher), marital status (married, or widowed/divorced/single), passive smoking (yes or no), body mass index (calculated as weight in kilograms divided by height in metres squared; <18.5, 18.5–24.9, or **⩾**25.0), walking duration (<1 or **⩾**1 h day^−1^), family history of cancer (yes or no), smoking status (never, former, or currently), number of cigarettes smoked per day (continuous variable), years of smoking (continuous variable), alcohol drinking (never, former, currently drinking <45.6 g day^−1^, or currently drinking **⩾**45.6 g day^−1^ ethanol), total energy intake per day (continuous variable), and daily consumption of soybean products, total meat, total fish, dairy products, total fruits, and total vegetables (for each food group, continuous variable), and consumption coffee (never or occasionally, 1–2 cups/day, or **⩾**3 cups/day).

c The total person-years does not equal to the sum of men and women because of rounding.

**Table 3:** Stratified multivariate adjusted hazard ratios and 95% confidence intervals for lung cancer incidence according to green tea consumption category, the Ohsaki Study

	**Green tea consumption, cups/day**				
	**<1**	**1 or 2**	**3 or 4**	**⩾5**	
**Characteristics**	**HR**	**HR (95% CI)**	**HR (95% CI)**	**HR (95% CI)**	***P* for trend** a
*Age*					
<60	1.00	0.80 (0.44–1.48)	1.09 (0.62–1.90)	1.11 (0.66–1.86)	0.52
⩾60	1.00	1.33 (0.85–2.06)	1.19 (0.77–1.86)	1.16 (0.77–1.74)	0.69
					
*Smoking status*					
Never	1.00	1.59 (0.68–3.69)	1.41 (0.61–3.26)	1.24 (0.54–2.84)	0.82
Former	1.00	0.91 (0.42–1.97)	0.94 (0.45–1.97)	1.22 (0.63–2.36)	0.55
Current	1.00	1.07 (0.64–1.77)	1.41 (0.88–2.27)	1.33 (0.85–2.07)	0.15
					
*Alcohol drinking*					
Never	1.00	1.38 (0.73–2.64)	1.10 (0.56–2.15)	1.18 (0.65–2.15)	0.82
Former	1.00	0.59 (0.15–2.35)	1.26 (0.41–3.84)	1.37 (0.52–3.60)	0.35
Current	1.00	1.11 (0.68–1.81)	1.34 (0.84–2.14)	1.23 (0.78–1.93)	0.31
					
*Education level*					
Junior high school or lower	1.00	1.23 (0.81–1.86)	1.06 (0.69–1.62)	1.13 (0.77–1.67)	0.69
High school or higher	1.00	0.83 (0.41–1.69)	1.11 (0.57–2.15)	1.01 (0.54–1.90)	0.76
					
*Walking duration, h day* ^−1^					
<1	1.00	0.96 (0.60–1.55)	0.90 (0.56–1.45)	0.84 (0.54–1.31)	0.42
⩾1	1.00	1.62 (0.92–2.86)	1.67 (0.95–2.93)	1.57 (0.93–2.65)	0.14
					
*Body mass index*					
<25	1.00	1.04 (0.69–1.56)	1.06 (0.71–1.58)	1.10 (0.76–1.58)	0.62
⩾25	1.00	1.31 (0.62–2.79)	1.22 (0.56–2.66)	0.97 (0.46–2.04)	0.81
					
*Family history of cancer*					
Yes	1.00	0.98 (0.52–1.85)	1.26 (0.70–2.28)	1.14 (0.66–1.97)	0.53
No	1.00	1.21 (0.79–1.84)	1.14 (0.74–1.75)	1.16 (0.78–1.72)	0.55
					
*Coffee*					
<1 cup/day	1.00	1.00 (0.60–1.67)	1.23 (0.78–1.96)	1.10 (0.71–1.70)	0.55
⩾1 cups/day	1.00	0.89 (0.49–1.61)	0.95 (0.52–1.76)	1.11 (0.64–1.92)	0.63

HR=hazard ratio; CI=confidence interval. For each stratified variable the multivariate HR has been adjusted for age (continuous variable), sex (men or women), education level (junior high school or lower, high school, or college/university or higher), marital status (married, or widowed/divorced/single), passive smoking (yes or no), body mass index (calculated as weight in kilograms divided by height in metres squared; <18.5, 18.5–24.9, or **⩾**25.0), walking duration (<1 or **⩾**1 h day^−1^), family history of cancer (yes or no), smoking status (never, former, or currently), number of cigarettes smoked per day (continuous variable), years of smoking (continuous variable), alcohol drinking (never, former, currently drinking <45.6 g day^−1^, or currently drinking **⩾**45.6 g day^−1^ ethanol), total energy intake per day (continuous variable), and daily consumption of soybean products, total meat, total fish, dairy products, total fruits, and total vegetables (for each food group, continuous variable), and consumption coffee (never or occasionally, 1–2 cups/day, or **⩾**3 cups/day).

a The *P*-values for the test of linear trend were calculated by treating the green tea consumption category as a continuous variable.
